# Prevalence and clinical impact of magnesium disorders in end-stage renal disease: a protocol for a systematic review

**DOI:** 10.1186/s13643-015-0063-x

**Published:** 2015-05-26

**Authors:** John Floridis, Asanga Abeyaratne, Sandawana William Majoni

**Affiliations:** Department of Nephrology, Division of Medicine, Royal Darwin Hospital, P.O. Box 41326, Casuarina, 0811 Australia; Flinders University and Northern Territory Clinical School, Royal Darwin Hospital Campus, Tiwi, 0810 Australia

## Abstract

**Background:**

Magnesium plays a key role in maintaining internal homeostasis through actions in the musculoskeletal, nervous, endocrine and cellular messenger systems. Renal excretion is the major route of magnesium elimination from the body. A positive magnesium balance would be expected in renal failure. However, a compensatory decrease in tubular reabsorption is expected to operate to maintain adequate urinary magnesium excretion even when glomerular filtration rate is very low.

Patients with end-stage renal disease and those on dialysis have impaired regulatory mechanisms, predisposing them to disturbances in magnesium levels. The effects of high or low magnesium can have deleterious health outcomes, which impact on the co-morbidities and outcomes of chronic renal disease. This systematic review aims to determine the prevalence and clinical outcomes of magnesium disorders in end-stage renal disease.

**Methods/Design:**

We will undertake a comprehensive search of various databases, MEDLINE, PubMED, EMBASE, Cochrane Library, Cochrane Collaboration, CIHNAL (Ebsco), Web of Science and Google Scholar, for observational studies and clinical trials on magnesium disorders in end-stage renal disease using key terms to identify papers for inclusion. Paper selection and data extraction (where appropriate) will be performed in duplicate on socio-demographic characteristics of participants, diagnosis of end-stage renal disease, magnesium levels, prevalence and clinical outcomes. An assessment of quality will be performed using a modified Newcastle-Ottawa Scale (NOS), including identification of any bias, which may influence findings. Data will be pooled together according to whether the studies were on pre-dialysis, hemodialysis or peritoneal dialysis participants. References from individual papers will also be screened as appropriate. Paper organisation and data extraction and analysis will take place using Microsoft Excel**®** and Stata version 13**®**.

**Discussion:**

This systematic review will represent a significant effort at pooling together information on prevalence and outcomes of magnesium disturbances amongst end-stage renal disease patients, which may guide further research and management of the disorders.

**Systematic review registration:**

PROSPERO: CRD42014014354

**Electronic supplementary material:**

The online version of this article (doi:10.1186/s13643-015-0063-x) contains supplementary material, which is available to authorized users.

## Background

Magnesium is the fourth most abundant cation in the body and has a role in bone metabolism, cardiovascular function, neurotransmission and multiple intracellular processes.

Magnesium levels in the body are tightly regulated by a combination of gastrointestinal and renal mechanisms to maintain homeostasis. Magnesium is primarily absorbed in the proximal small intestine. Excretion occurs via ultrafiltration of free magnesium through the glomerulus. Once filtered, a substantial amount may be reabsorbed, predominately in the ascending loop of Henle [1]. Under normal conditions, both the intake and excretion of magnesium may be altered to maintain a normal range of total magnesium between 0.65 and 0.74 mmol/L [2].

Patients affected by chronic kidney disease (CKD) experience altered magnesium homeostasis, primarily through reduced renal excretion, predisposing them to developing hypermagnesaemia. Whilst there is a lack of tubular reabsorption, this does not adequately compensate for the reduced ultrafiltration. In patients with end-stage renal disease who require haemodialysis or peritoneal dialysis, dialysate fluid contains a variable amount of magnesium, which may be higher, lower or equivalent to the target range of normal magnesium levels [2]. Patients receiving dialysis with low concentrations of magnesium may be predisposed to developing hypomagnesaemia, due to diffusional movements from the circulation. Additionally, patients with CKD may be hypomagnesaemic for other reasons, including the use of proton pump inhibitors, malnutrition or co-mordities such as alcoholism [3].

Hypomagnesaemia has been observed to increase cardiovascular risk in patients with chronic kidney disease [4]. The mechanism is complex, however low magnesium levels facilitate endothelial dysfunction, and consequently, arterial intimal thickening, arterial calcification and oxidative damage, all of which can accelerate atherosclerosis [5–9]. It is suspected that magnesium also plays a role in the altered bone metabolism in chronic renal failure; however, further research is required to further establish this relationship [2].

This systematic review specifically aims to analyse evidence on the prevalence of magnesium disturbances in patients with end-stage renal disease. We aim to pool together data from observational studies and clinical trials to assess trends in magnesium levels and effects on outcomes such as cardiovascular co-morbidities, for example, ischaemic heart disease, vascular calcification, peripheral vascular disease, diabetes, hypertension and hypercholesterolemia.

## Methods/Design

### Protocol and registration

The methods and design of this systematic review are based on recommendations from the Reporting Items for Systematic Reviews and Meta-Analyses (PRISMA-P) statements and the Meta-analysis of Observational Studies in Epidemiology (MOOSE) [10, 11]. PRISMA requires a description of the eligibility criteria using the PICOS (the participants, interventions, comparisons, outcome(s) and study design of the systematic review) reporting system. This review will include observational studies and clinical trials, hence we will use both the PICOS and, in accordance with the MOOSE guidelines, the eligibility criteria by means of the participants/population, exposure(s), comparator(s)/control, outcome(s) and study design (PECOS). The patient population are patients with end-stage renal disease and those on haemodialysis or peritoneal dialysis. The exposures will be any factors, which we feel may impact serum magnesium levels, including, but not limited to, dialysate concentration, co-morbidities, concurrent proton pump inhibitor use and use of magnesium supplementation. Outcomes will be serum magnesium levels, in addition to mortality (adjusted for confounders), and impact on associated co-morbidities, for example, glycaemic control or dyslipidaemia. The protocol is registered with the International Prospective Register of Systematic Reviews (Registration Number: CRD42014014354).

### Objectives

The aim of this systematic review is to assess the prevalence of magnesium disorders in patients with end-stage renal disease: pre-dialysis, and those on peritoneal dialysis and haemodialysis. Furthermore, we hope to assess the associated impact on outcomes such as cardiovascular disease and other outcomes.

### Inclusion and exclusion criteria

The population of interest will be all patients with end-stage renal disease, pre-dialysis and those requiring regular haemodialysis or peritoneal dialysis. Cross-sectional, cohort, clinical trials, case–control and observational studies will be included. Individual case reports will be excluded. Studies may arise from any geographical or socioeconomic location but must be published in English.

### Study selection

Studies assessing prevalence of magnesium disorders and outcomes in end-stage renal disease will be selected. Search results will be imported and stored into Endnote software. Two reviewers will independently screen titles and abstracts for relevance and consideration into a provisional list. Individual lists of articles will then be assessed independently by each reviewer for potential inclusion.

After a defined time period, the two reviewers will meet and reach a consensus about which articles are to be included. A third party investigator will discuss any disagreement until a consensus is reached. Upon completion, a flowchart will be prepared, as outlined in Additional file 1, which will include numerical values of accepted and rejected studies throughout this process.

### Search strategy

We will conduct the search by formulating a database containing published studies in peer-reviewed journals, addressing the prevalence and outcomes of magnesium disorders in end-stage renal disease patients. A systematic search of MEDLINE via Ovid, PubMED, Excerpta Medica Database (EMBASE), Cochrane Library, Cochrane Collaboration, Cochrane Database of Systematic Reviews (CDSR), Nursing and Allied Health Literature (CIHNAL) (Ebsco), Web of Science and Google Scholar will be undertaken. We will examine publications from 1966 until 2014. Search terms will include controlled vocabulary and text-words. Terms to be searched in isolation and combination will include the following: “prevalence”, “magnesium disorders”, “hypomagnesaemia”, “hypermagnesaemia”, “magnesium disturbance”, “magnesium”, “mortality”, “all cause mortality”, “cardiovascular mortality”, “all cause mortality”, “survival”, “long term survival”, “carotid intima media thickness”, “vascular calcification”, “renal disease”, “chronic kidney disease”, “end-stage renal disease”, “dialysis”, “peritoneal dialysis”, “haemodialysis”, “cardiovascular”, “ischaemic heart disease”, “vascular disease”, “diabetes”, “hypertension” and “hypercholesterolaemia”. Terms will be expanded to take into account spelling differences of keywords between different countries and journals. See Additional file 2 for more details, which summarises the search terms for MEDLINE.

The search will be performed in close cooperation with an experienced librarian. Articles for selection must be peer reviewed, full text and written in English. Reference lists of individual papers will also be reviewed where appropriate and relevant to the clinical question.

### Data extraction

Papers and any appropriate data will be categorised and standardised using Microsoft Excel**®** and Stata version 13**®**. One reviewer will categorise papers, extract and input any relevant data from the final list of studies, whilst the second reviewer will validate data. The following data will be extracted:

1. Publication details
2. Study design
3. Study participant details (baseline characteristics)
4. Stage of end-stage renal disease: pre-dialysis, haemodialysis or peritoneal dialysis
5. Co-morbidities
6. Use of proton pump inhibitors
7. Composition of dialysate fluid, specifically magnesium and calcium concentration
8. Data for outcome measures: serum magnesium levels
9. Data for additional clinical outcome measures, including, but not limited to glycaemic control, lipid profiles, blood pressure, or arterial calcification
10. Limitations including any bias which affect quality of the paper

### Quality assessment

Two reviewers, using a standardised approach, will independently assess the quality of papers. This process will follow the initial selection of papers as outlined previously. Any discrepancies will be resolved by discussion, including a third reviewer where consensus is not reached.

To increase the robustness of the quality assessment for this review, we will use the Newcastle-Ottawa Scale (NOS) for the quality assessment of non-randomised studies. [12]. We will modify it for clarity of scoring the studies for quality [13]. The risk of bias will be independently assessed by two reviewers for each of the included studies using the modified NOS, specific to the context of this review. This will include seven questions spread across four domains for evaluating: methods for selecting study participants (selection bias), methods to control for confounding factors (performance bias), statistical methods (detection bias) and methods for measuring exposure and outcome variables (information bias). Risk of bias is measured on a scale of 0 (high risk of bias) to 3 (low risk of bias). A specific description with examples of both high and low bias is provided in Additional file 3. In the modification of the NOS, items regarding selection of participants (representativeness of sample) and ascertainment of outcome (objective versus subjective measures) were retained, whilst other items relating to the comparability of groups and adequate follow-up for cohort and case–control studies were removed as these are not directly applicable to current review. Categories that emphasise statistical methods, confounding effects and reporting of data to ensure that bias in methodology are minimised will be assessed. These scales will be used to measure the risk of bias on a per study basis or categorized by domain to develop a general conclusion about the sources of bias in the studies included in this review [13]. Quality scores will be presented in a table. An additional file shows this in more detail [see Additional file 4].

### Data synthesis

The number of papers included and excluded during the systematic review process will be clearly presented in a flowchart for clear transparency, as outlined in Additional file 1. It is anticipated that there will be heterogeneity amongst papers reviewed, in their design and dataset. Evidence tables will be used to summarise relevant data extracted from eligible studies. However, papers selected will be presented in summary table for clear interpretation and application to the systematic review. Subgrouping of data will be performed by end-stage renal disease status (pre-dialysis, peritoneal dialysis or haemodialysis). A summary of the prevalence of magnesium disorders will be provided in a narrative synthesis according to patient details, stage of chronic kidney disease, dialysis modalities, and co-morbidities as well as the predictors of magnesium disorders wherever possible. Limitations of the studies will be discussed in detail. In observational papers, the main outcome of interest will be presented, based on the nature of the study.

The review will also comprise subgroup analysis by end-stage renal disease status and draw upon any associations between variables.

## Discussion

Magnesium disorders in people with end-stage renal disease may be more common than is currently known. It is not clear in many guidelines whether treatment to correct magnesium disorders is warranted. The strength of this review is that it will to clearly establish the prevalence of magnesium disorders in ESRD patients. Whilst previous literature has highlighted the concerns around magnesium disturbances in this population, results are variable with many associations in place, based on indirect *in vitro* and *in vivo* studies. Furthermore, whilst hypomagnesaemia has been acknowledged as a concern due to impact on clinical outcomes, there is still no consensus about how to manage this electrolyte imbalance in clinical practice—with or without magnesium supplementation in people with ESRD.

### Limitations

Study inclusion will involve judgements from both reviewers. Whilst this process will be undertaken independently to minimise bias, we acknowledge that, where duplication of studies exist, author bias can still occur.

### Dissemination

The results of the systematic review will be published in peer-reviewed journals and presented at conferences where appropriate.

## Additional files

Additional file 1:
**Flow chart to summarise the search strategy.** The flowchart illustrates the proposed method of systematically reviewing literature, which will include numerical values upon completion. Additional file 2:
**Search strategy—MEDLINE via Ovid.** The search strategy outlines the keywords, which will be used in MEDLINE via Ovid, however a homogenous approach will be incorporated in other search engines to ensure all relevant literature is captured as part of the review. Additional file 3:
**Modified Newcastle-Ottawa Scale (NOS) For Quality Assessment of the Studies Included in the Systematic Review.** The modified NOS will be applied to all literature reviewed to assess for quality. Complete scores will be available in the completed systematic review, which allow conclusions from various studies to be weighted accordingly. Additional file 4:
**Proposed table of studies reviewed, incorporating modified NOS assessment.** This table will be the proposed format in which the studies are listed in the systematic review to allow for a clear and concise presentation of the relevant literature.

## Abbreviations

ESRD: End-stage renal disease
CKD: Chronic kidney disease
NOS: Newcastle-Ottawa Scale
