# Supplementary material for: Prevalence and clinical impact of magnesium disorders in end-stage renal disease: a protocol for a systematic review
Source: Syst Rev. 2015 May 26;4:76. doi: 10.1186/s13643-015-0063-x (PMC4446798; doi:10.1186/s13643-015-0063-x)
Supplement: Additional file 1: — Flow chart to summarise the search strategy. The flowchart illustrates the proposed method of systematically reviewing literature, which will include numerical values upon completion. [file 13643_2015_63_MOESM1_ESM.docx]

**Additional File One: Flow Chart to Summarize the Search Strategy**

Total number included in the systematic review, **n=**

Studies excluded on further assessment based on inclusion criteria screening, **n=**

Studies added after search on references from full text screening, **n=**

Studies included for full text screening, **n=**

Studies excluded by exclusion and inclusion criteria screening, **n=**

Studies retrieved for abstract screening, **n=**

Studies excluded during initial screen, **n=**

Studies excluded after duplication screening**, n=**

Studies for title screening, **n=**

Search databases: Primary MEDLINE via Ovid, PubMED, Exceprta Medica Database (EMBASE), Cochrane Library, Cochrane Collaboration, Cochrane Database of Systematic Reviews (CDSR), Nursing and Allied Health Literature (CIHNAL) (Ebsco), Web of Science and Google Scholar-studies for title search
