# Supplementary material for: Prevalence and clinical impact of magnesium disorders in end-stage renal disease: a protocol for a systematic review
Source: Syst Rev. 2015 May 26;4:76. doi: 10.1186/s13643-015-0063-x (PMC4446798; doi:10.1186/s13643-015-0063-x)
Supplement: Additional file 2: — Search strategy—MEDLINE via Ovid. The search strategy outlines the keywords, which will be used in MEDLINE via Ovid, however a homogenous approach will be incorporated in other search engines to ensure all relevant literature is captured as part of the review. [file 13643_2015_63_MOESM2_ESM.docx]

**Additional File Two: Search strategy - MEDLINE via Ovid**

1. Hypomagnesemia or hypomagnesaemia.tw
2. Hypermagnesemia or hypermagnesaemia.tw
3. 1 or 2
4. Magnesium.tw
5. Disorder or disturbances.tw
6. 4 and 5
7. Prevalence.tw
8. Renal disease*.tw
9. Chronic kidney disease*.tw
10. End stage renal disease*.tw
11. 8 or 9 or 10
12. Dialysis*.tw
13. Haemodialysis or hemodialysis.tw
14. Peritoneal dialysis.tw
15. 12 or 13 or 14
16. 11 or 15
17. 3 and 7
18. 3 and 16
19. 6 and 7
20. 6 and 16
21. Cardiovascular disease*.tw
22. Ischaemic heart disease or Ischemic heart disease*.tw
23. Dyslipidaemia or dyslipidemia.tw
24. hypercholesterolaemia or hypercholesterolemia.tw
25. 23 or 24
26. Vascular disease.tw
27. Vascular calcification
28. Diabetes*.tw
29. Hypertension*.tw
30. (3 or 6) and 16 and 21
31. (3 or 6) and 16 and 22
32. (3 or 6) and 16 and 25
33. (3 or 6) and 16 and 26
34. (3 or 6) and 16 and 27
35. (3 or 6) and 16 and 28
36. (3 or 6) and 16 and 29
