# Supplementary material for: Prevalence and clinical impact of magnesium disorders in end-stage renal disease: a protocol for a systematic review
Source: Syst Rev. 2015 May 26;4:76. doi: 10.1186/s13643-015-0063-x (PMC4446798; doi:10.1186/s13643-015-0063-x)
Supplement: Additional file 4: — Proposed table of studies reviewed, incorporating modified NOS assessment. This table will be the proposed format in which the studies are listed in the systematic review to allow for a clear and concise presentation of the relevant literature. [file 13643_2015_63_MOESM4_ESM.docx]

| **Authors, Year, Country** | **Study design, methods** | **Recruitment and eligibility methods** | **Prevalence rates of low Mg** | **Modified Newcastle- Ottawa Scale** | | | **Limitations of study** |
| --- | --- | --- | --- | --- | --- | --- | --- |
|  |  |  |  | **Domain** | **Question** | **Score** |  |
|  |  |  |  | 1 | 1 |  |  |
|  |  |  |  | 2 | 2 |  |  |
|  |  |  |  |  | 3 |  |  |
|  |  |  |  | 3 | 4 |  |  |
|  |  |  |  |  | 5 |  |  |
|  |  |  |  | 4 | 6 |  |  |
|  |  |  |  |  | 7 |  |  |

**Additional File Four: Proposed table of studies reviewed, incorporating modified NOS assessment**
